# Intravenous Application of Pulsed Radiofrequency–4 Case Reports

**DOI:** 10.5812/aapm.10242

**Published:** 2013-07-01

**Authors:** Alexandre Teixeira, Menno E. Sluijter

**Affiliations:** 1 Clínica de Dor, Porto, Portugal; 2 Centers for Pain Medicine, Swiss Paraplegic Center, Nottwil, Switzerland

**Keywords:** Pulsed Radiofrequency Treatment, Allostasis, Immune System, Inflammation, Neoplasms

## Abstract

It has been suggested that PRF might possibly have an effect on the immune cells. We considered using the intravenous route to apply PRF in conditions that are caused by an unresolved immune action or connected to allostatic load, implicating an abnormally reacting immune system to obtain a systemic effect that could possibly be an additional tool in treating some of these conditions. These manuscript reports four cases that illustrate the wide variety of conditions where this new technique might be helpful.

## 1. Introduction

Pulsed radiofrequency is nowadays a well-recognized clinically harmless technique for chronic pain treatment (1). It was initially introduced as a potentially more tolerable and safer procedure to replace continuous radiofrequency thermolesions as in trigeminal neuralgia (2) and chronic facet joint pain (3). It is also used as a primary choice treatment when the collateral damage produced by the application of continuous radiofrequency turns it into an inappropriate treatment as in postamputation phantom pain (4). But new avenues for its applications were developed in unexpected ways as describe by Sluijter and Imani (5) in 2008, when intra-articular Pulsed Radiofrequency (PRF) was introduced (6), it was suggested that PRF might possibly have an effect on immune cells. This application is now long past its initial stage. Good long term results have been published on the atlanto-axial join (7). This was a novel aspect of PRF. It then occurred to us that there is quite a list of conditions that are either caused by an unresolved immune action, such as chronic infections, or connected to allostatic load (8), implicating an abnormally reacting immune system. If PRF could be applied in a different way causing a systemic effect, it could possibly be an additional tool in treating some of these conditions. We considered using the intravenous route. We based this on the work of Nordenstrom (9), who used weak direct currents intravascularly to treat malignant tumors. He reports that the electrical resistivity of the blood vessel wall is much higher than the resistivity of blood. This implies that the vascular tree can to some extent be used to propagate electric fields. We investigated the method briefly but we abandoned our plans at that time. Then in 2009 there was a coincidence. A colleague who knew about our brief efforts was diagnosed with an advanced lung carcinoma. On his own initiative he requested treatment with intravenous PRF (see patient #1). He eventually had a complete and lasting remission. We then got some requests from relatives and friends for treatment of diverse conditions. Out of compassion we have honored a few of them, from patients who were at the end of the road of conventional treatment. We here report four cases. We applied PRF by puncture of an antecubital vein with a 23 G 60 mm XE needle (NeuroTherm, Wilmington, and Mass) with a 5 mm active tip. As a dispersive electrode we used a 5 x 5 cm skin electrode as it is used in TENS. This electrode was applied on the contralateral side on a location that was adapted to the condition to be treated. For example, in patient #3 (long standing depression), the electrode was placed over the contralateral vagus nerve. We applied 60 V for 15 minutes. The duty cycle was 4 x 10 msec. The impedance ranged from 300 to 600 Ohm. The procedure was uneventful. Some patients noticed a faint pulsing sensation if a small diameter vein had been used. There have been no side effects or complications.

## 2. Case presentation

### 2.1. Patient #1. Complete Remission of an NSC Lung Carcinoma

A 59-year-old plastic surgeon was diagnosed with a lung tumor on 31 March 2009, when he was examined for persistent upper dorsal spinal pain, refractory to TENS and physiotherapy. He had smoked 20 cigarettes a day for the last 40 years. He felt healthy except for a lack of energy in the last two weeks and he had a good performance status. His tumor was classified as a Stage IV right lung, poorly differentiated, large cell adenocarcinoma. It was a T2 (1,6 cm nodule in the right parahilar region), N2 (4,3x3, 1x5,3 cm bulky conglomerate of subcarinal nodes) and M1 (left suprarenal metastasis of 2,7 cm diameter). The carcinoembryonic antigen (CEA) had a high value of 203,4 ng/ml. He was scheduled for four cycles of platinum doublet based chemotherapy with cysplatinum and premetrexed at every 21 days, to start on May 11. This patient requested treatment with intravenous PRF. This was done on April 11, 2009, 3 weeks before the start of chemotherapy. He reported a subjective feeling of wellbeing starting during treatment and continuing thereafter. Over the 3 weeks following treatment, his dorsal pain with an NRS score of 8 gradually disappeared completely. Also, on a second PET-CT on May 8 2009, before the start of chemotherapy, there was no tumor progression of the parahilar nodule. He was then treated with chemotherapy and later with chemoradiotherapy, and the adrenal metastasis was removed endoscopically. After the chemoradiotherapy, ending on October 9, there was no more active treatment. He kept doing well and a PET-CT made on February 3, 2010 failed to show any evidence of disease. The last checkup was done in May 2012. He was again tumor free and the CEA was 7 ng/ml.

### 2.2. Comment

A spontaneous complete remission of an NSC lung carcinoma is extremely rare (10). We have been able to find only one single case of adenocarcinoma of the lung stage I, with a complete remission (11). A complete and durable result of chemotherapy and chemoradiotherapy is equally rare (12). In this case, the complete remission of pain and the stable image on the scan in the period before chemotherapy was started suggest that possibly PRF treatment may have played an additional role in the therapeutic process.

### 2.3. Patient #2. α 1 anti-Trypsin Deficiency

A 50-year-old construction engineer had to stop working at the age of 31 because of increasing dyspnea and coughing. A diagnosis of α 1 anti-trypsin deficiency was made. He was not dyspneic in rest, but he had very little tolerance for exercise. He could walk up a flight of stairs but he had to do it slowly.What bothered him most was the occurrence of intercurrent infections. Especially in the winter season this happened frequently. There was then increased and productive coughing, moderate fever and a feeling of sickness. It often took him 2 to 4 weeks to recover and the next infection might occur very soon afterwards. He requested treatment with intravenous PRF. He was treated on March 13, 2011. He has had no intercurrent infections since, and he was very pleased with the result. To put it in his own words: “I know that I have the disease, but I’m not sick anymore”. On his urgent request the treatment was repeated as a precautionary measure on October 17, 2011 and on March 11, 2012. His pulmonologist saw him last on July 5, 2011 for a checkup. He was very pleased with the physical findings, on auscultation the lungs were dry and the lung function showed no deterioration compared to a test in 2001. He encouraged him to continue the PRF treatment.

### 2.4. Patient #3 - Exacerbation of a Long Standing Depression

A 42 years old female nurse had a history of major depressive disorder since the age of 19 years. Since then she had been under continuous psychiatric treatment, involving continuous anti-depressive medication. Lately she was medicated with clozazolan 2 mg and venlafaxine 75 mg daily. Mid December 2011 she developed an acute depressive episode. That was not a new phenomenon; acute crises had been recurrent, occurring without any warning, provocation or specific time schedule. They had sometimes required the need for hospitalization, but they had been responsive to medication in the past. This time the dose of clozazolan and venlafaxine were doubled and trazodone 150 mg was additionally prescribed. The medication was not efficacious and she also became too drowsy. After consultation with her psychiatrist she was treated with intravenous PRF on 11 April 2011. The dispersive electrode was placed over the contralateral carotid artery. Before the procedure hs-CRP was 9,4 mg/l. On the day after treatment she reported that she felt more alert but that her mood was unchanged. After the first week she started improving progressively. After 2 weeks she felt no longer depressed and she started reducing the medication to venlafaxine 75mg. At 4 weeks the hs-CRP was again determined, it had dropped to 3,8 mg/l. In June, two months after treatment, she stopped taking all medication – for the first time in 23 years - and she started working again. She has been doing very well for 9 months. Then suddenly on 11/01/2012 she had a recurrent acute episode. She reinitiated the medication and she requested a new intravenous PRF treatment. This was done on 25/01/2012. She is now doing well again without medication.

### 2.5. Patient #4 – Ischemic Stroke in Left Middle Cerebral Artery Territory

A 46 year old, healthy female secretary, a relative of one of the authors (AT), had a sudden episode of apraxia and right hemiplegia on 30/10/2009, while she was resting at home. She was immediately transferred to the hospital where she arrived unconscious. A diagnosis of ischemic stroke in the left middle cerebral artery encompassing the insula, frontal and temporal lobes was established and appropriate treatment was implemented. No cardiac or carotid vessel disease was identified and the etiology was undetermined. She survived the acute episode with a right hemiparesis, aphasia and apraxia. A rehabilitation program was initiated with physiotherapy and speech therapy on the fifth day post stroke. She was discharged on 18/11/2009, having by then fully recovered from the hemiplegia and partially from the apraxia. She continued the rehabilitation program as an outpatient. Over the first month there was some progress in recovery from the apraxia and the aphasia, but then she stopped improving further and she abandoned the program. At the time her aphasia was classified as a mild to moderate Broca’s aphasia. In January 2010 she requested intravenous PRF treatment. At that time her situation was as follows. Verbal expression was limited to a small repertoire vocabulary and short sentences. Auditory comprehension was well preserved but only if her interlocutor talked at a slow rate. She could gesticulate and write. Her reading capacity was also limited to words and sentences; she was unable to read paragraphs. She was alert and cooperative but anxious and mildly depressed due to her clinical condition. In making the decision we considered that spontaneous improvement had come to an end. We expected that it might be possible to improve her condition by reducing the perilesional inflammatory component with a positive effect on the neuroplasticity and that possibly her mood would improve. She was treated on January 1, 2010. A high sensitivity CRP was taken immediately before the procedure and repeated 1week after. It had dropped from 1,05 mg/l to 0,33 mg/l. The immediate outcome was a subjective feeling of well-being. Thereafter the symptomatology progressively improved, starting in the first week and continuing for 8 months. By the end of April 2010 she restarts cooking and driving her car. By then she was capable of reading magazines. Her speech repertoire had also improved and she could maintain a conversation with several interlocutors if they talked at a slow pace. She and her husband referred that she improved the most from July to September. After that period there was stabilization and she asked for a repeat treatment. This was done on 29/12/2010 and the result was a further improvement of the aphasia. She and her husband commented that she improved more in the 4 months after the second treatment than in all the previous year. Her sexual life, which had come to a stop, returned to normal in this period. She resumed her job with some limitations in June 2011. Actually she lives an almost normal life now with a mild expressive aphasia.

## 3. Discussion

These case reports illustrate the wide variety of conditions where this new technique might be a helpful tool. The non-malignant cases had one common denominator. They all had inflammation as part of their pathology. Although our data are anecdotal we could not escape one impression. In successful cases the immune system seems to switch to a new, more active equilibrium that provides a reduction of inflammation and a more efficient surveillance. This was most obvious in the patients with lung disease, but the other treated conditions have a relationship with sterile inflammation. Depression is not a primary inflammatory condition, but inflammation is thought to be both a precipitating factor in vulnerable individuals and a perpetuating factor impeding recovery (13). Stroke causes a massive sympathetic outflow, and this in turn causes a change of immune cells, in this case the invariant NKT-cells, to a more regulatory phenotype (14). The resulting immune suppression, favoring sterile inflammation, plays an important role in the pathology. The CRP level correlates independently with the outcome (15). In case report #4 parasympathetic stimulation may have played an additional role (16) since the groundplate was placed over the carotid artery. As for cancer patients, we decline from comment at this point. Some patients are doing quite well, but we have to wait for end results. This report is not an encouragement to the reader to start adding more casuistics to this series. We strongly feel that progress can only be made by initiating proper, prospective studies. This may be a difficult road to travel because it requires the cooperation – or, even better, the initiative – of other disciplines that are less familiar with PRF.
